# Supplementary material for: Loss of the Arp2/3 complex component ARPC1B causes platelet abnormalities and predisposes to inflammatory disease
Source: Nat Commun. 2017 Apr 3;8:14816. doi: 10.1038/ncomms14816 (PMC5382316; doi:10.1038/ncomms14816)
Supplement: Supplementary Information — Supplementary Figures, Supplementary Tables, Supplementary Notes and Supplementary Reference. [file ncomms14816-s1.pdf]

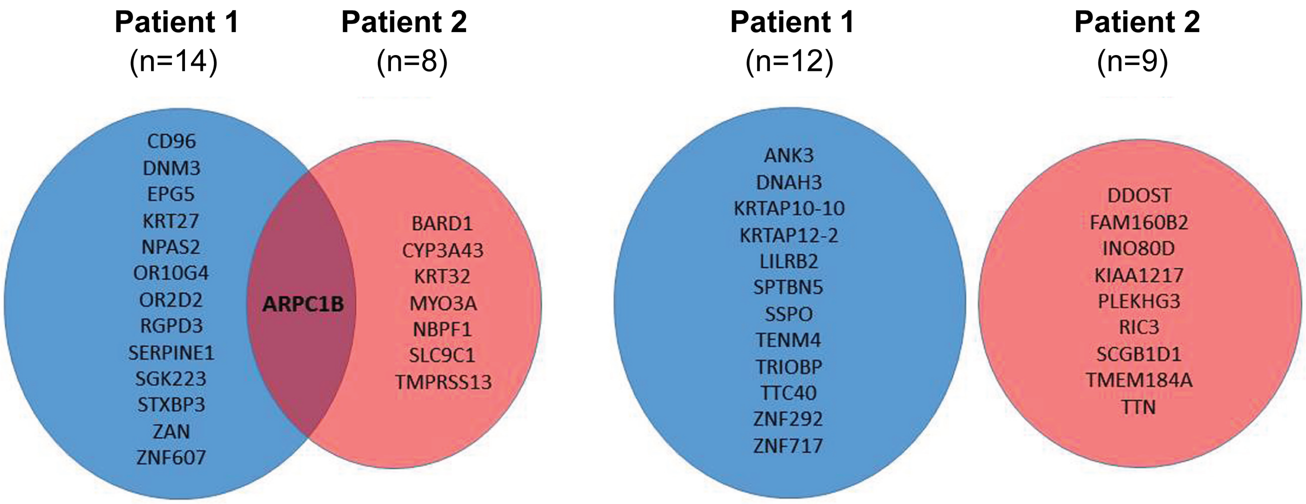

Supplementary Figure 1 Venn diagrams of genes from Patient 1 and Patient 2. Rare coding variants (n=number of variants; minor allele frequency, maf<0.01) are shown that fit an autosomal recessive (left) or compound heterozygous (right) inheritance pattern.

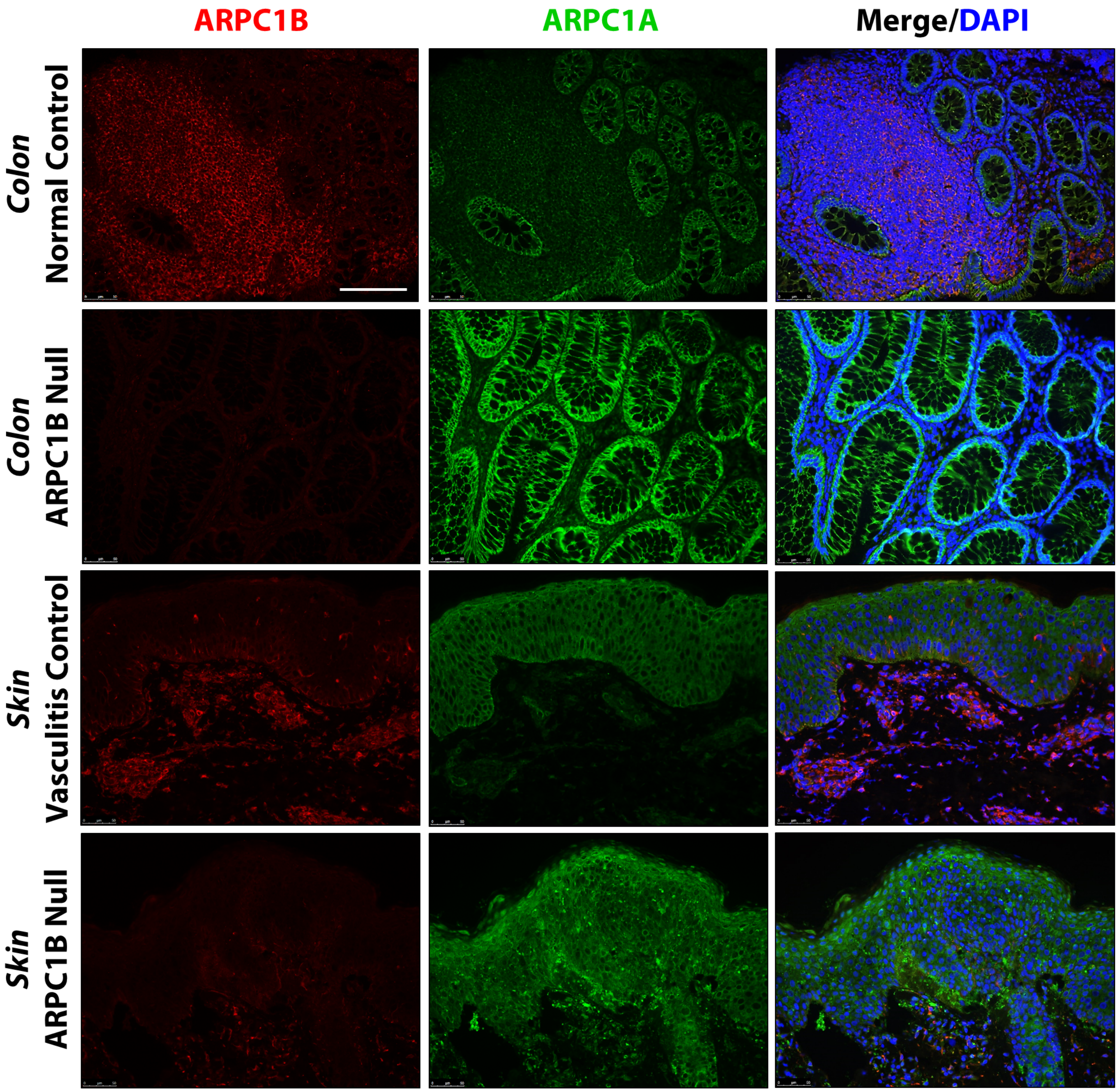

Supplementary Figure 2 ARPC1A and ARPC1B expression in colon and skin of Patient 1 compared to controls. Immunofluorescence microscopy of colon (top two rows) and skin (bottom two rows) sections from Patient 1 (ARPC1B null, second and fourth row) compared to controls (first and third row). The sections were stained with antibodies against ARPC1B (first column, red), ARPC1A (second column, green) and with DAPI (4',6-diamidino-2-phenylindole) for DNA (blue) where the images were also merged (Merge/DAPI, third column). Magnification 20X (Bar = 100 μm).

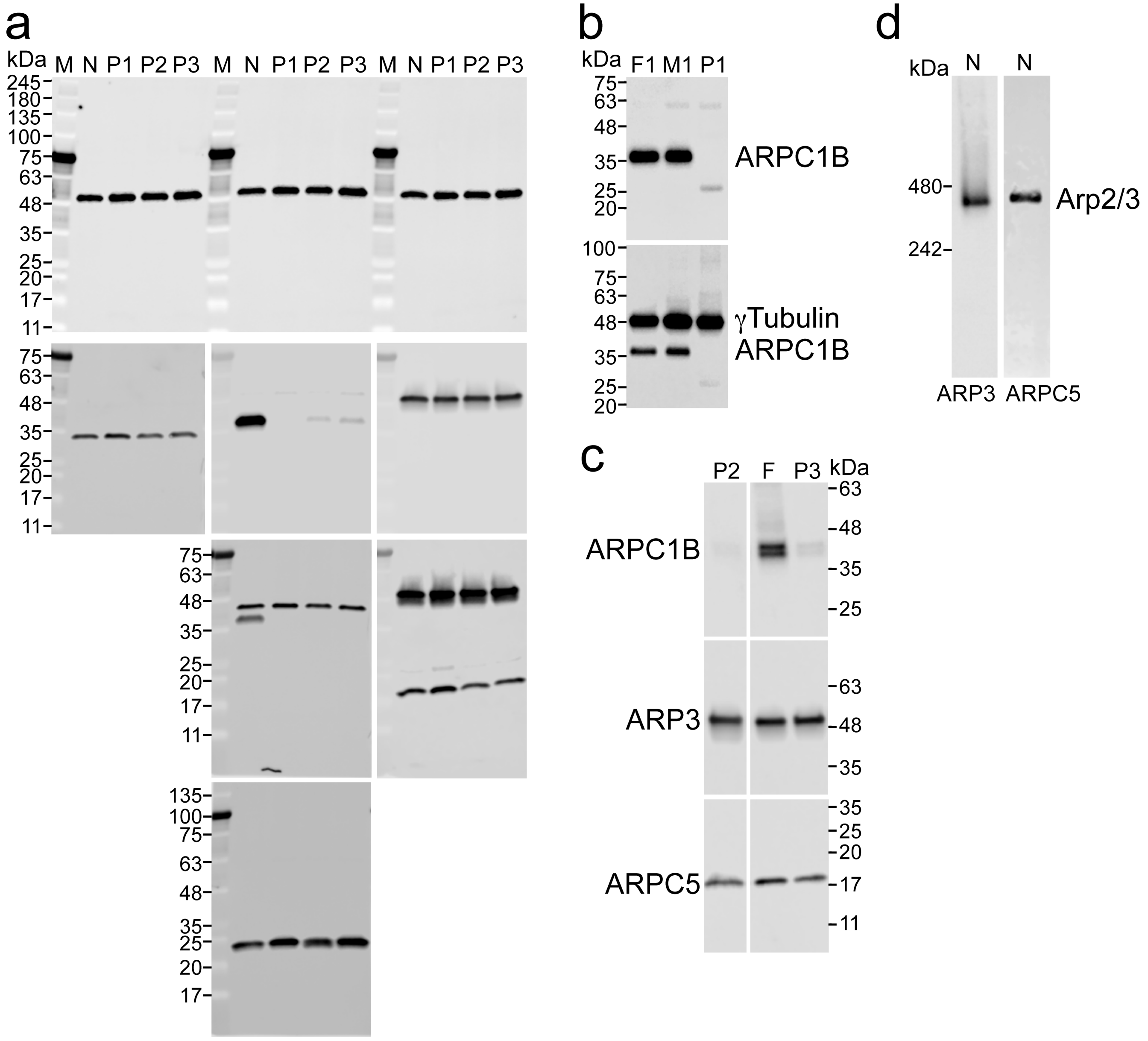

Supplementary Figure 3 Immunoblot analysis of patients, family members and normal controls. (**a**) Summary of immunoblot analysis of normal (N) and ARPC1B mutation patient (P1, P2, P3) platelet lysates for expression of Arp2/3 component proteins. Samples were run in triplicate on a 4-20% SDS-PAGE gel, and the resulting blot was divided and probed sequentially with rabbit antibodies specific for ARPC2 (left), ARPC1B, ARP2 and ARPC3 (middle), or ARP3 and ARPC5 (right); protein sizes are shown in kilodaltons (kDa) in the corresponding marker (M) lane. All blots were probed with a mouse monoclonal antibody to gamma tubulin as a protein loading control (upper blot). (**b**) Immunoblot of platelet lysates from Patient 1 (P1), his father (F1) and his mother (M1) probed for ARPC1B and gamma tubulin. (**c**) Immunoblot of platelet lysates from Patient 2 (P2), Patient 3 (P3) and their father (F) probed for ARPC1B, ARP3 and ARPC5. (**d**) Arp2/3 detected by blue native gel electrophoresis and immunoblotting in normal platelet lysate. The native Arp2/3 complex was detected by probing for ARPC1B (Fig. 2d), and for other components including ARP3 (left) and ARPC5 (right).

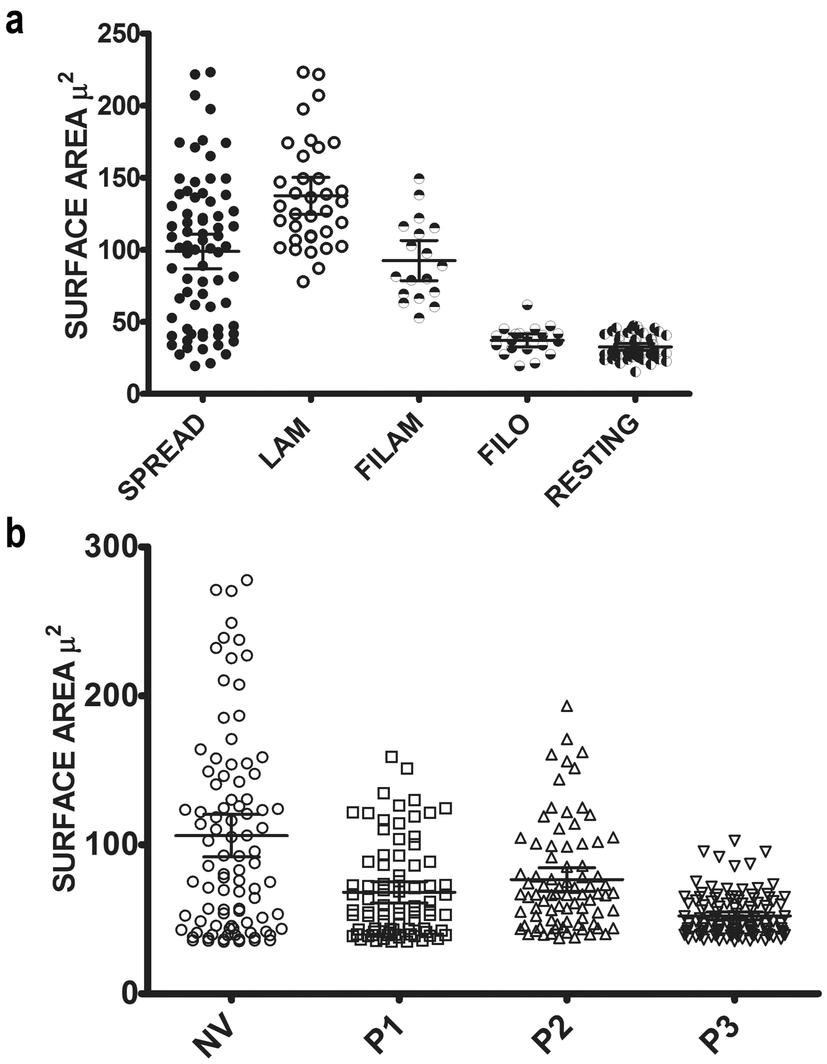

Supplementary Figure 4 Surface area and morphology of platelets spread on fibrinogen. (**a**) Platelets from a normal donor were prepared and imaged as in Fig. 7, and cell surface areas were obtained using Volocity software. The total distribution of all spread cells (SPREAD; n = 73 cells) was broken down into 4 morphological classes based on visual scoring: fully lamellipodial cells (LAM, n = 73), filopodial cells (FILO, n = 20), cells showing both filopodia and lamellipodia (FILAM, n = 18) and unspread/resting cells (RESTING, n = 49). These morphological classes were observed to have different surface area distributions, with lamellipodial cells being largest by mean surface area. (**b**) Surface area distributions of spread platelets (defined as having a surface area >35 μm^2) from a normal donor (NV) and patients 1-3 (P1, P2, P3) show ARPC1B-deficient cells had significantly smaller mean surface areas relative to normal (unpaired two-tailed t-test p<0.0007; n = 79 normal, 81 Patient 1, 80 Patient 2, 58 Patient 3). Distributions of patient platelets also lacked the larger lamellipodial morphology class (pooled results for experiment done twice for normal, Patients 1 and 2, once for patient 3). Means and 95% confidence intervals shown by bars in both graphs.

Supplementary Table 1 ARPC1B mutation identification and analysis

Patient	SNP	Exon	Mutation Position			ExAC MAF	Prediction						
			Genomic (GRCh37.p13)	cDNA	Protein		SIFT	Polyphen2	MutationTaster	MutationAssessor	FATHMM	GERP++ RS	PhyloP
1	Novel	4	g.98985760->CT	NM_005720.3: c.269_270dupCT	NP_005711.1: p.Val91Trpfs*30	Novel	N/A	N/A	N/A	N/A	N/A	N/A	N/A
2/3	Novel	4	g.98985806C>T	NM_005720.3: c.314C>T	NP_005711.1: p.Ala105Val	Novel	Damaging	Possibly Damaging	Disease causing	Predicted non-functional (Low)	Tolerated	4.51	0.47118
	rs147238850	7	g.98988805G>A	NM_005720.3: c.712G>A	NP_005711.1: p.Ala238Thr	0.0001483	Tolerated	Benign	Polymorphism	Predicted non-functional (Neutral)	Tolerated	2.84	0.23485

Supplementary Table 2 Arp2/3 complex components in platelets

Protein	Size (kD)	Abundance (ppm)	Rank (of 6429)
**ARP2**	45	1419	130
**ARP3**	47	1527	114
**ARPC1A**	42	87	1627
**ARPC1B**	41	1503	124
**ARPC2**	34	2931	51
**ARPC3**	21	1260	150
**ARPC4**	20	1058	173
**ARPC5**	16	1585	112

Relative amounts of Arp2/3 complex components in human platelets determined by mass spectrometry. (from Kim et al, 2014[1])

Supplementary Table 3 ARPC1A and ARPC1B in human tissues and cells

Tissue/Cell	ARPC1A	ARPC1B
**Whole body**	32	245
**Lymph node**	9	1232
**B cell**	24	788
**Monocyte**	33	815
**Gut**	231	225
**Spinal cord**	241	118
**Brain**	312	27

Abundance (ppm) of ARPC1A and ARPC1B in various human tissues and cells (source: PaxDb Protein Abundance Across Organisms, http://pax-db.org; most primary data from Kim et al., 2014[1])

Supplementary Note 1 Clinical information

Patient 1 was born at term after a normal pregnancy and delivery to parents of South Asian heritage who are second cousins. At one month of age he suffered a neck abscess and meningitis, complicated by septic shock and a cerebrovascular accident (superior sagittal sinus thrombosis with left posterior parietal-infarction) due to *Staphylococcus aureus* infection. Thrombocytopenia was identified at this time. At five months he had an episode of bloody diarrhea attributed to rotavirus that resolved. At nine months he developed a marked papular and nodular rash. A skin biopsy showed subepidermal vesiculobullous lesions with superficial perivascular infiltrates including mast cells and lymphocytes, consistent with vasculitis. A bone marrow aspirate conducted at 10 months appeared normal with adequate megakaryopoiesis (Fig. 8). Repeated bone morrow examination showed increased eosinophils.

He had low albumin levels and suffered painless rectal bleeding at age 3. Initial colonic biopsies showed inflammatory bowel disease characterized by mild crypt distortion and inflammation. Repeat biopsy showed severe eosinophilic infiltration in the colon and rectum that responded well to steroids and sulfasalazine. Cervical lymphadenopathy was evaluated with biopsies that found reactive plasma cell hyperplasia and sinus histiocytosis, and no evidence of neoplasia. At age 4 he developed a painful rash that flared periodically. Skin biopsy showed leukocytoclastic vasculitis with depositions of IgG, IgM and C3. He also experienced mild joint limitation in the elbows and wrists with slow progression to bony erosions. At this stage he was found to have a positive ANA at 1/640 with an unclear pattern, and an intermittently positive ANCA (atypical p-pattern with anti-myeloperoxidase and anti-pr3 negative). Anti-dsDNA, anti-sm, anti-RNP, anti-RO and anti-La were all negative. His predominant clinical feature and ongoing morbidity was related to severe cutaneous vasculitis with a variable response to immunosuppression using disease-modifying anti-rheumatic drugs including methotrexate, azathioprine, mycophenolate mofetil (MMF) and intravenous immunoglobulin (IVIG). Disease flares were treated by short-term systemic corticosteroids with good response. A trial of anti-tissue necrosis factor therapy for severe flare was ineffective, with disease control only achieved with high-dose intravenous corticosteroids.

In summary, this patient's infectious history shows repeated invasive infections since the first episode of meningitis, including pneumonia at ages 2 and 5, cellulitis and *S. aureus* infections of surgical wounds after cervical lymph node biopsies at age 3 and 4. He continued to have *S. aureus* infections, including a finger abscess at age 6 and recurrent blepharitis of both eyes. He never had opportunistic infections. At most recent follow-up at 9 years old he was maintained on antimicrobial prophylaxis, monthly IVIG infusions and daily MMF, and showed mild clinical symptoms (skin vasculitis), persistent microthrombocytopenia and biochemical markers of inflammation at upper limits of normal.

Patient 2 was born at term after a normal pregnancy to non-consanguineous parents of Scottish heritage. He had extensive cradle cap at 8 weeks of age and developed eczematous appearing rash on his hands which quickly followed by the development of target lesions, purpura and swelling. Simultaneously he developed subconjunctival hemorrhage. Skin biopsy revealed leukocytoclastic vasculitis with deposition of IgM and C3 and he was ANA and ANCA positive. Brain MRI and an angiography showed no evidence of vasculitis. He was treated with corticosteroid and IVIG, and recently at age 9 months was introduced to methotrexate as a steroid sparing agent.

Patient 3 is the older brother of Patient 2 and was identified by genetic screening of the *ARPC1B* mutation (Supplementary Table 1) in this family. The patient did not have clinical disease at the time of testing but platelet, biochemical and immunological work-up suggest subclinical manifestation of *ARPC1B*-deficiency (Table 1). He was born at term after a normal pregnancy. His past medical history was largely unremarkable. He had an eczematous-like rash intermittently since a few weeks of life that responded well to topical hydrocortisone with no superinfection, although these skin lesions did leave residual hypopigmentation. His infectious history found only one episode of clinically diagnosed

pneumonia at age 1-2 years old and impetigo of the face at age 6, both treated with oral antibiotics. He had a clinical diagnosis of asthma with one related emergency department visit at age 6. He had no symptoms of rheumatological disease but he had persistently elevated CRP and he screened positive for ANCA.

Evaluation of the immune system. Patient 1 and 2 had frequent lymphocytosis and eosinophilia while Patient 3 also had elevated eosinophils (Table 1). Immunophenotyping demonstrated the number of circulating $CD19^+$ B cells were markedly increased in both Patient 1 and 2, and at the upper limit of normal in Patient 3. This correlated with a marked increase in serum immunoglobulins IgA and IgE in all three patients. Normal numbers of circulating $CD3^+$ T cells and $CD4^+$ cells with a low $CD8^+$ T cell count were found in Patient 1. Evaluations of T cell output by measuring T-cell receptor excision circles (TRECs) revealed normal production of T cells. *In vitro* responses to mitogens appeared normal in all three patients. Antibody responses to vaccination including tetanus toxoid and pneumococcus appeared normal.

Supplementary Reference

1. Kim MS*, et al.* A draft map of the human proteome. *Nature* **509**, 575-581 (2014).
